# Headache classification and automatic biomarker extraction from structural MRIs using deep learning

**DOI:** 10.1093/braincomms/fcac311

**Published:** 2022-11-26

**Authors:** Md Mahfuzur Rahman Siddiquee, Jay Shah, Catherine Chong, Simona Nikolova, Gina Dumkrieger, Baoxin Li, Teresa Wu, Todd J Schwedt

**Affiliations:** School of Computing and Augmented Intelligence, Arizona State University, Tempe, AZ, USA; ASU-Mayo Center for Innovative Imaging, Tempe, AZ, USA; School of Computing and Augmented Intelligence, Arizona State University, Tempe, AZ, USA; ASU-Mayo Center for Innovative Imaging, Tempe, AZ, USA; ASU-Mayo Center for Innovative Imaging, Tempe, AZ, USA; Department of Neurology, Mayo Clinic, Phoenix, AZ, USA; Department of Neurology, Mayo Clinic, Phoenix, AZ, USA; Department of Neurology, Mayo Clinic, Phoenix, AZ, USA; School of Computing and Augmented Intelligence, Arizona State University, Tempe, AZ, USA; ASU-Mayo Center for Innovative Imaging, Tempe, AZ, USA; School of Computing and Augmented Intelligence, Arizona State University, Tempe, AZ, USA; ASU-Mayo Center for Innovative Imaging, Tempe, AZ, USA; ASU-Mayo Center for Innovative Imaging, Tempe, AZ, USA; Department of Neurology, Mayo Clinic, Phoenix, AZ, USA

**Keywords:** headache classification, headache biomarkers, migraine, post-traumatic headache, persistent post-traumatic headache

## Abstract

Data-driven machine-learning methods on neuroimaging (e.g. MRI) are of great interest for the investigation and classification of neurological diseases. However, traditional machine learning requires domain knowledge to delineate the brain regions first, followed by feature extraction from the regions. Compared with this semi-automated approach, recently developed deep learning methods have advantages since they do not require such prior knowledge; instead, deep learning methods can automatically find features that differentiate MRIs from different cohorts. In the present study, we developed a deep learning-based classification pipeline distinguishing brain MRIs of individuals with one of three types of headaches [migraine (*n* = 95), acute post-traumatic headache (*n* = 48) and persistent post-traumatic headache (*n* = 49)] from those of healthy controls (*n* = 532) and identified the brain regions that most contributed to each classification task. Our pipeline included: (i) data preprocessing; (ii) binary classification of healthy controls versus headache type using a 3D ResNet-18; and (iii) biomarker extraction from the trained 3D ResNet-18. During the classification at the second step of our pipeline, we resolved two common issues in deep learning methods, limited training data and imbalanced samples from different categories, by incorporating a large public data set and resampling among the headache cohorts. Our method achieved the following classification accuracies when tested on independent test sets: (i) migraine versus healthy controls—75% accuracy, 66.7% sensitivity and 83.3% specificity; (2) acute post-traumatic headache versus healthy controls—75% accuracy, 66.7% sensitivity and 83.3% specificity; and (3) persistent post-traumatic headache versus healthy controls—91.7% accuracy, 100% sensitivity and 83.3% specificity. The most significant biomarkers identified by the classifier for migraine were caudate, caudal anterior cingulate, superior frontal, thalamus and ventral diencephalon. For acute post-traumatic headache, lateral occipital, cuneus, lingual, pericalcarine and superior parietal regions were identified as most significant biomarkers. Finally, for persistent post-traumatic headache, the most significant biomarkers were cerebellum, middle temporal, inferior temporal, inferior parietal and superior parietal. In conclusion, our study shows that the deep learning methods can automatically detect aberrations in the brain regions associated with different headache types. It does not require any human knowledge as input which significantly reduces human effort. It uncovers the great potential of deep learning methods for classification and automatic extraction of brain imaging–based biomarkers for these headache types.

## Teaser

Rahman Siddiquee *et al.* report a deep learning-based classification method that automatically discovered imaging biomarkers specifically associated with migraine (for example, caudate, caudal anterior cingulate and superior frontal), acute post-traumatic headache (for example, lateral occipital, cuneus and lingual) and persistent post-traumatic headache (for example, cerebellum, middle temporal and inferior temporal).

## Graphical Abstract

**Graphical abstract:**
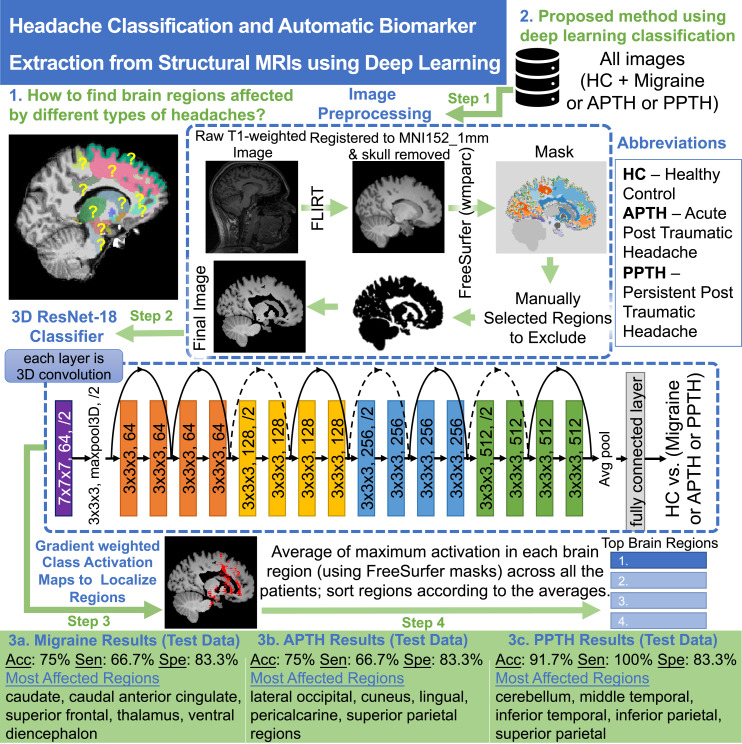

## Introduction

Machine-learning (ML) techniques are now widely used for medical image analyses. Many ML methods have been developed to identify structural, functional and molecular biomarkers from neuroimages such as MRI and PET. Imaging biomarkers have been used for classification and prognostications of neurodegenerative diseases such as Alzheimer’s disease^1,2^ and Parkinson’s disease.^3^ The use of imaging-based biomarkers in the headache field to study migraine,^4,5^ post-traumatic headache (PTH) and other headache types is still in an early developmental phase. The potential value of ML approaches and a summary of results from ML studies in the headache field were presented in a 2020 article by Messina and Filippi.^6^ Traditional ML approaches rely heavily on pre-selecting features as input to an assumed inference model, hence requiring domain knowledge as a prior. While the pre-selection of brain regions based on prior knowledge is a targeted approach, it limits the discovery of other regions that might be important as disease biomarkers.

The recent advances in deep learning (DL) methods have made it possible to learn both feature extraction and inference models from the imaging data directly, and such models have been shown to outperform traditional ML methods.^7^ Drawing upon the success of DL in the computer vision field, neuroimaging research has also benefited from DL for the study of neurodegenerative diseases, potentially contributing to earlier diagnosis, disease staging, prognosis and therapeutic development.^8–11^ However, development of brain imaging–based DL models for migraine and other headache types has been limited. We contend that one reason for DL methods being underutilized relates to the availability of relatively small data sets. Compared with computer vision research which is often supported by a large volume of images (e.g. at the million scale) for model training, DL-based neuroimaging research typically: (i) has much smaller numbers of imaging samples and (ii) the data set is often imbalanced, e.g. having more samples from healthy controls (HCs) than from headache and migraine patients. Both the size and the balance of the data set are crucial to building a robust DL model.^12^

In the present study, we developed a DL-based technique for classification of participants with migraine, acute PTH (APTH) or persistent PTH (PPTH) versus HC followed by an automated feature extractor pipeline that identified brain regions affected by migraine and PTH. Migraine, a primary headache, affects about 12% of the general population, is a substantial cause of disability and is a common headache type for which individuals seek care in the outpatient clinic.^13–15^ PTH is among the more common secondary headache types, is the most common symptom immediately following mild traumatic brain injury (mTBI), is often persistent and typically has symptoms that overlap with those of migraine.^16–20^ In the present study, we aimed to develop accurate DL-based classification models for these common and impactful primary and secondary headache types. In our method, we resolved the challenges of (i) a relatively small data set by incorporating HCs from a public data set and (ii) data imbalance by oversampling headache samples to match the HCs. For classification, we used a 3D ResNet-18^21^ model which has proved effective in computer vision tasks.^22–26^ The ResNet architecture supports building deep neural networks to capture different levels of details in an image without suffering from typical difficulties associated with increasing depth of the network. For medical imaging tasks, they have been used in classification and early diagnosis of Alzheimer’s disease,^27–29^ differentiating benign and malignant tumours,^30^ subtype classification of heamorrhages^31^ and skin lesion detection.^32^ These research studies show that ResNet models can automatically learn highly discriminative feature representations of respective medical imaging data and perform classification with significant performances. Herein, we used a neuroimaging data-driven approach to develop and test classification models and discover brain regions most contributing to such models for Migraine, APTH and PPTH using ResNets.

## Materials and methods

### Data sets

In the present study, we utilized five data sets collected by investigators at the Mayo Clinic Arizona and one public data set (information extraction from images, IXI). The Mayo Clinic data sets included brain MRIs from 296 individuals, including HC (*n* = 104), Migraine (*n* = 95), APTH (*n* = 48) and PPTH (*n* = 49). The IXI public data set included MRIs from 428 HCs. For each data set, the total number of participants with their age and sex distribution is summarized in Table 1.

**Table 1:** Summary of all the data sets

Data set	No. of participants	Age (mean ± SD)	Sex
1	34 migraine	39.8 ± 12.8	3 M, 31 F
2	28 migraine	38.5 ± 10.5	8 M, 20 F
	25 HC	38.0 ± 10.4	8 M, 17 F
3	33 migraine	41.2 ± 11.3	13 M, 20 F
	49 PPTH	38.1 ± 10.5	32 M, 17 F
	38 HC	38.2 ± 9.6	21 M, 17 F
4	10 APTH	37.3 ± 12.7	3 M, 7 F
5	38 APTH	42.7 ± 13.3	16 M, 22 F
	41 HC	38.4 ± 12.5	16 M, 25 F
6 (IXI, public)	428 HC	42.4 ± 13.0	196 M, 232 F

#### Institutional data sets (Data sets 1–5): participant enrolment and characteristics

All studies performed at the Mayo Clinic were approved by the Mayo Clinic Institutional Review Board. Participants were identified from the Mayo Clinic in Arizona and the Phoenix VA healthcare system. All participants provided written informed consent prior to participation. All study participants were men and women between the ages of 19 and 65 years. Individuals with abnormal brain imaging findings, according to usual clinical interpretation, were excluded. Women who were pregnant were excluded from study participation.

*Migraine*: Participants were diagnosed with episodic migraine or chronic migraine, without and/or with aura, according to the most recent version of the International Classification of Headache Disorders (ICHDs) available at the time the participant was enrolled (ICHD-3 beta or ICHD-3.^33,34^

*Post-traumatic headache*: APTH and PPTH attributed to mTBI were diagnosed according to the most recent version of the ICHDs available at the time the participant was enrolled (ICHD-3 beta or ICHD-3).^33,34^ Those with any history of moderate or severe TBI were excluded. Those with APTH were enrolled between 0 and 59 days post-mTBI. Participants with PPTH were enrolled at any time after the development of PPTH.

*Healthy controls*: HC were excluded if they had any history of headache other than tension-type headache on three or fewer days per month.^35–37^

##### Image acquisition

All participants studied at Mayo Clinic Arizona were imaged on one of two 3 tesla Siemens (Siemens Magnetom Skyra, Erlangen, Germany) scanners using a 20-channel head and neck coil. Anatomical T_1_-weighted images were acquired using magnetization prepared rapid image acquisition gradient echo (MPRAGE) sequences. Image acquisition parameters for T_1_-weighted images are repetition time (TR) = 2400 ms; echo time (TE) = 3.03 ms; flip angle (FA) = 8°; voxel size = 1 × 1 × 1.25 mm.^3^

#### Public data set (Data set 6): participant enrolment and characteristics

To support the DL effort which often requires large data sets, we used IXI^38^ data set containing MRIs from HC. The images were acquired between June 2005 and December 2006. In total, 277 male and 342 female participants were enrolled. The participants were between 20.0 and 86.3 years of age with an average age of 49.4 and a standard deviation of 16.7. To match the age distribution of our participants with headache, we only included participants between 20 and 64 years of age. Therefore, our final cohort contains 196 male and 232 female subjects with an average age of 42.4 and a standard deviation of 13.0.

The subjects from the IXI data set were obtained from three different hospitals in London: Hammersmith Hospital, Guy’s Hospital, and Institute of Psychiatry. The data set contains T_1_, T_2_, proton density, magnetic resonance angiography and diffusion tensor imaging for each subject. We used only T_1_-weighted images. Hammersmith Hospital used a Philips 3T system for acquiring the images. This system used the following T_1_ parameters: TR = 9.6, TE = 4.6, acquisition matrix = 208 × 208, FA = 8. Guy’s Hospital used a Philips 1.5T system with T_1_ parameter TR = 9.813, TE = 4.603, FA = 8°. The Institute of Psychiatry used a GE 1.5T system. The T_1_ imaging parameters for this scanner were not released at the time of writing this manuscript.

### Image preprocessing

The MRI scans for all six data sets were stored in Neuroimaging Informatics Technology Initiative format. In our preprocessing pipeline, first, we performed skull stripping of raw images that removed the non-brain areas from the scans using the Brain Extraction Tool^39^ available within the FSL package (Wellcome Center, University of Oxford, UK). These skull-stripped images were then aligned to the MNI152 template with 1 mm resolution using the linear registration tool FLIRT.^40^ We then performed White Matter Parcellation (wmparc) on the images using FreeSurfer. We overlayed the parcellation masks on the images and removed 14 regions from the images not relevant to our study. These regions are left vessel, right vessel, right lateral ventricle, left lateral ventricle, right unsegmented white matter, left unsegmented white matter, left choroid plexus, right choroid plexus, left inferior lateral ventricle, right inferior lateral ventricle, fourth ventricle, third ventricle, cerebral spinal fluid (CSF) and optic chiasm. We also set all voxels marked unknown (mostly regions outside the brain) in the parcellation mask to zero which helps to remove motion artefact, if any, from the images. Finally, we used the resultant images to train a 3D ResNet-18 classifier.

### Headache classification and automatic biomarker extraction

In the present study, we performed three classification tasks: (i) migraine versus HC; (ii) APTH versus HC; and (iii) PPTH versus HC. We randomly split the data set into three: training set, validation set and blind testing set (Table 2). The validation is to identify the best DL model and the blind test is to demonstrate the robustness and generalization of the model on unseen data. Since our training data set was highly imbalanced, we sampled the migraine/APTH/PPTH images to match the number of samples in HC cohort during training. It is a common approach in traditional ML approach for imbalanced learning and known as oversampling. As introduced earlier, we used a DL-based 3D ResNet model (ResNet-18) for the classification tasks. Once the classifier was trained, in each task, we applied the GradCAM^41–43^ method (a well adopted method in computer vision research) on the trained ResNet-18 to extract brain regions that contributed to the classification according to the DL model. This is to support clinical interpretations for potential clinical utilization.

**Table 2:** Design of three classification experiments

Experiments	Training	Validation	Blind test
Migraine versus HC	83 migraine 520 HC^a^	6 migraine 6 HC^a^	6 migraine 6 HC^a^
APTH versus HC	36 APTH 520 HC^a^	6 APTH 6 HC^a^	6 APTH 6 HC^a^
PPTH versus HC	37 PPTH 520 HC^a^	6 PPTH 6 HC^a^	6 PPTH 6 HC^a^

APTH, acute post-traumatic headache; HC, healthy controls; PPTH, persistent post-traumatic headache. ^a^ Please note that across all the splits and tasks the HC subjects are the same.

**Figure 1:**
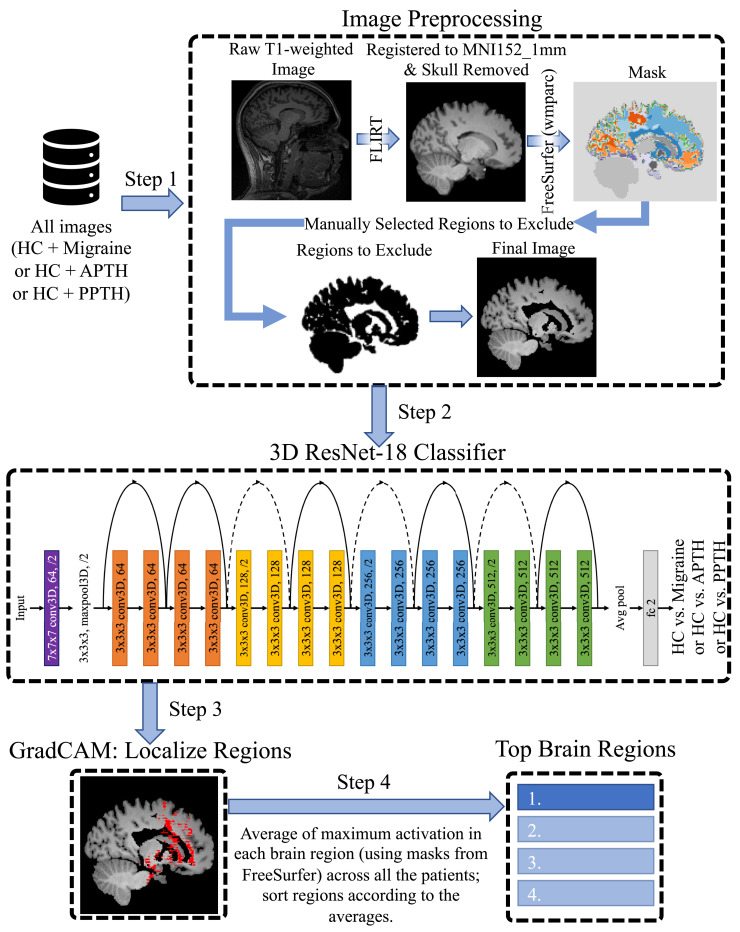
**The research pipeline.** For each task, we remove the skull and remove regions outside the brain, as well as some brain regions unnecessary to our study. Finally, we use a 3D ResNet-18 to classify HC versus each of the three headache types.

The training process is visualized in Fig. 1.

### Statistical analyses

Statistical analyses and drawings were performed by software environment Office 365 (Microsoft). We used two-tailed, two-sample unequal variance *t*-test. *P*-values <0.05 were considered statistically significant.

## Results

### Migraine classification

Average age (migraine 39.9 ± 11.6 years, HC 41.6 ± 12.7 years, *P* = 0.2) did not differ between groups. However, there were significantly more females in the migraine group (migraine 71/95 or 74.74% female) than in the HC group (HC 291/532 or 54.70% female, *P* = 0.0001). Participants with migraine averaged 15.3 ± 7.4 headache days per 28 days. Of these patients, 37 had episodic migraine and 58 had chronic migraine, and on average, they had migraine for 21 ± 12.8 years. Presence of migraine with aura attacks were reported by 49/95 participants. Medications that could be taken for migraine prevention were being used by 27/95 participants.

**Figure 2:**
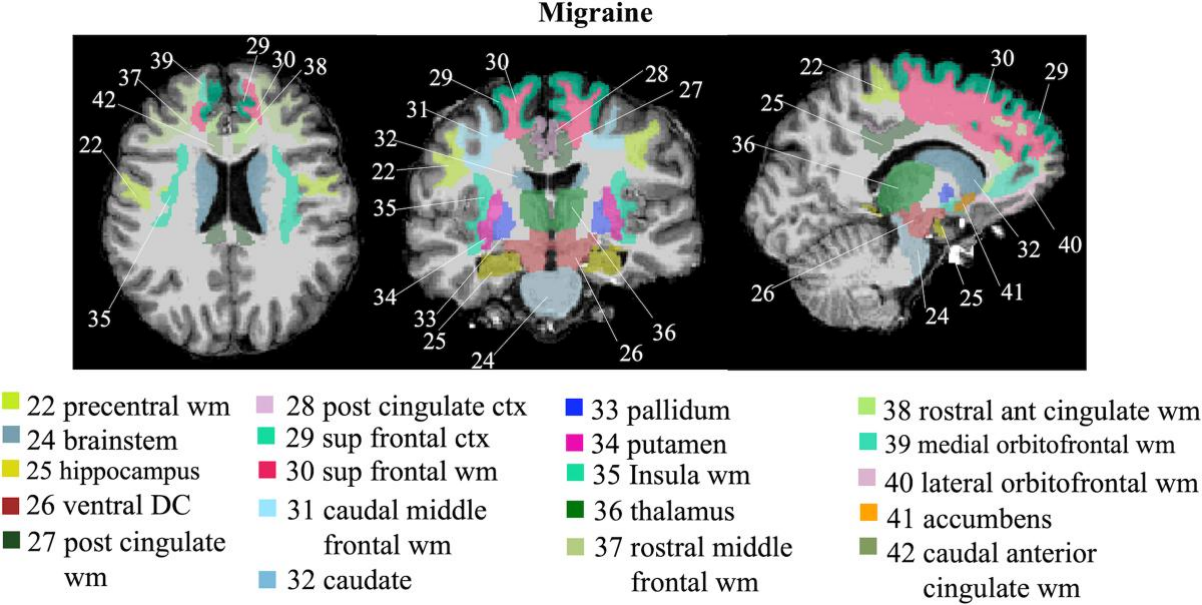
**Visualization of brain regions most contributed to migraine classification.** ant, anterior; ctx, cortex; dc, diencephalon; post, posterior; sup, superior; wm, white matter.

Our method achieved 83.33 and 75% accuracy overall on validation and unseen testing data, respectively. Specifically, for validation, our method achieved 83.33% sensitivity and 83.33% specificity. For the blind testing, our method achieved 66.67% sensitivity and 83.33% specificity. The brain regions that most contributed to migraine classification included: caudate, caudal anterior cingulate white matter, superior frontal (white and grey matter), thalamus, ventral diencephalon (includes hypothalamus, subthalamic nucleus, lateral and medical geniculate nucleus, red nucleus, substantia nigra and mamillary bodies), posterior cingulate (white and grey matter), medial orbitofrontal white matter, pallidum, accumbens area, putamen, rostral anterior cingulate white matter, lateral orbitofrontal white matter, brain stem, rostral middle frontal white matter, insula white matter, hippocampus, caudal middle frontal white matter and precentral white matter. These brain regions are visualized in Fig. 2.

### Acute post-traumatic headache classification

Age (APTH 41.5 ± 13.3 years, HC 41.6 ± 12.7 years, *P* = 1) and sex (APTH 29/48 female, HC 291/532 female, *P* = 0.4) did not differ between groups. Those with APTH had onset of APTH an average of 24.5 ± 14.5 days at their time of imaging, and they had headaches on an average of 76.3±29.6% of days since APTH onset. Medications that can be used for headache prevention were being taken by 5/48 participants. mTBIs were due to motor vehicle accidents (*n* = 20), falls (*n* = 21) and direct hits to the head (*n* = 7).

**Figure 3:**
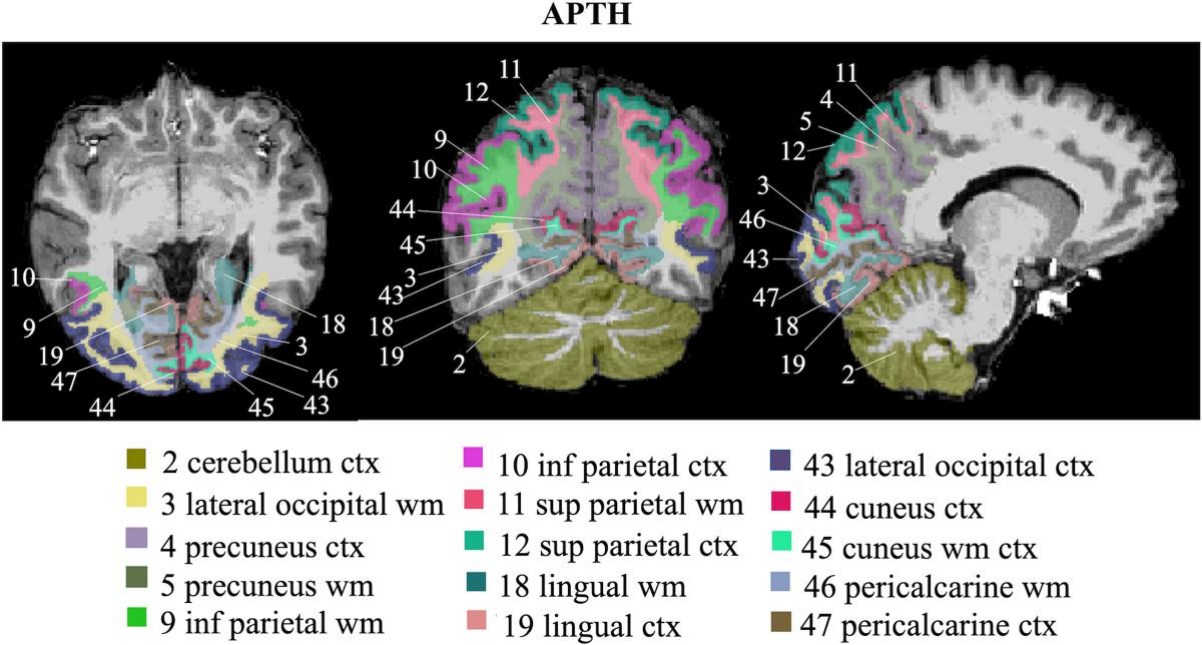
**Visualization of brain regions that most contributed to APTH classification.** ctx, cortex; inf, inferior; sup, superior; wm, white matter.

The classification model achieved 91.67% accuracy on validation (83.33% sensitivity, 100% specificity) and 75% accuracy on unseen testing data (66.67% sensitivity, 83.33% specificity). The brain regions that most contributed to APTH classification included: lateral occipital (white matter and grey matter), cuneus (white matter and grey matter), lingual (white matter and grey matter), pericalcarine (white matter and grey matter), superior parietal (white matter and grey matter), precuneus (white and grey matter), inferior parietal (white matter and grey matter) and cerebellum grey matter. These brain regions are visualized in Fig. 3.

### Persistent post-traumatic headache classification

Age did not differ between the two groups (PPTH 38.2 ± 10.6 years, HC 41.6 ± 12.7 years, *P* = 0.04), while sex did (PPTH 17/49 female, HC 291/532 female, *P* = 0.007). Those with PPTH averaged 15.3 ± 7.4 headache days per month, and they had PPTH for an average of 10.6 ± 8 years. Medications that can be used for headache prevention were being taken by 23/49 participants. mTBIs were attributed to sport related injuries (*n* = 8), falls (*n* = 12), motor vehicle accidents (*n* = 7) and blast injuries (*n* = 22).

**Figure 4:**
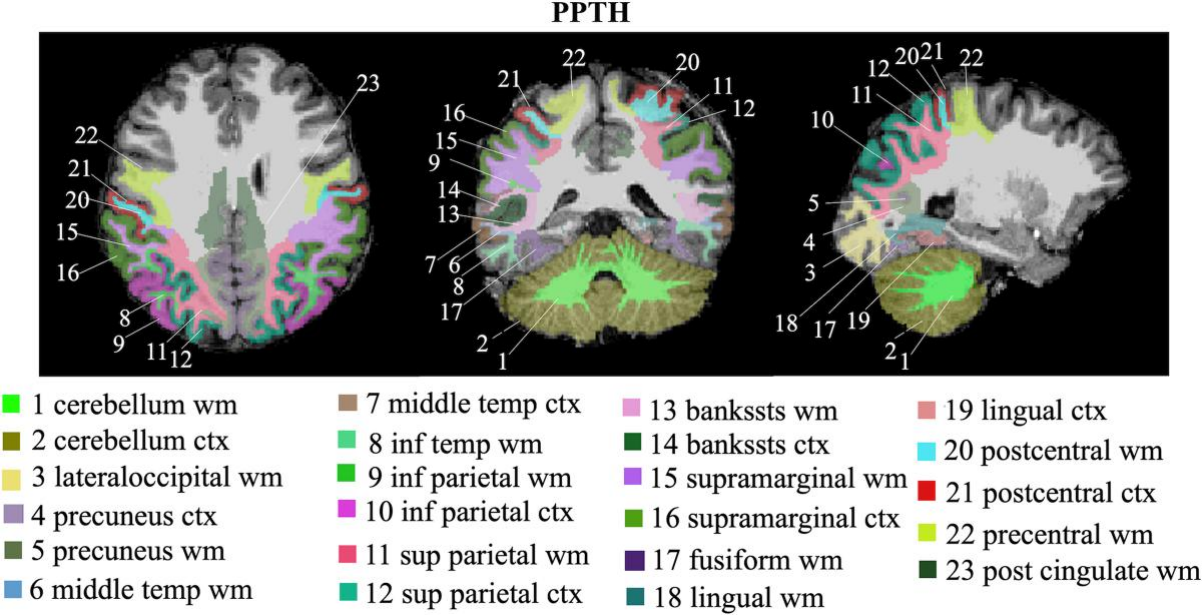
**Visualization of brain regions that most contributed to PPTH classification.**; bankssts, banks of superior temporal sulcus; ctx, cortex; inf, inferior; post, posterior; sup, superior; temp, temporal; wm, white matter.

The classification model achieved 66.67% accuracy on validation (83.33% sensitivity, 50% specificity) and 91.67% accuracy on unseen testing data (100% sensitivity, 83.33% specificity). The brain regions that most contributed to PTH classification included: cerebellum (white and grey matter), middle temporal (white matter and grey matter), inferior temporal white matter, inferior parietal (white and grey matter), superior parietal (white and grey matter), banks of superior temporal sulcus (bankssts) (white and grey matter), precuneus (white and grey matter), supramarginal (white and grey matter), fusiform white matter, lingual (white matter and grey matter), lateral occipital white matter, postcentral (white and grey matter), precentral white matter and posterior cingulate white matter. These brain regions are visualized in Fig. 4.

## Discussion

This study used a DL approach for the development and testing of brain MRI-based classification models for migraine, APTH and PPTH. MRI data from several of our studies and a publicly available data set were combined to achieve a large sample size and to enhance the generalizability of the classification models. Using previously unseen brain MRIs (i.e. MRIs not included during model training) for testing, the classification tasks differentiating those with headache from HCs achieved overall accuracy of 75% for migraine, 75% for APTH and 92% for PPTH. Furthermore, the brain regions most contributing to the classifications were identified.

Our group and others have previously developed brain MRI-based classification models for migraine and PTH. This study is unique due to the DL approach, the large number of MRIs included during model development, validation and testing, and the determination of model accuracy using an independent set of brain MRIs that were not included during model development or validation. Despite the assumption that classification accuracy would be lower when using an independent testing set, our classification accuracies in this study meet or exceed those reported in prior publications which have typically used leave-one-out cross-validation methods.

Prior publications that have developed imaging-based classification models for migraine or PTH have used structural (e.g. regional volumes, cortical thickness) and functional connectivity measures. ML methods have typically been used for model development. These approaches require a priori identification of brain regions of interest for inclusion in the classification models. The DL approach used in our study does not require pre-selection of regions, reducing the chance of missing important features, while still allowing identification of brain areas that most contributed to headache classification.

Prior brain imaging-based classification models for migraine have achieved leave-one-out classification accuracies ranging from about 68 to 84% for differentiating migraine from HC. A study using measures of cortical thickness, surface area and volume for 68 brain regions classified migraine from HCs with 68% accuracy.^4^ A resting-state functional connectivity study including 33 regions of interest had 81% accuracy for differentiating migraine from HCs.^44^ Studies combining functional connectivity and structural data have reported classification accuracies of 83–84%.^45,46^ The accuracy of the DL-based classification model in our study (75%) falls within the previously reported ranges, is superior to prior models that included only measures of brain structure and is likely to be a better estimate of true accuracy, since it was determined using a completely independent test set. Brain areas found to most contribute to classification in our study included several that were found to be important in prior studies, such as Schwedt *et al.*^4^ These regions are caudal anterior cingulate, caudal middle frontal, posterior cingulate, insula, medial orbitofrontal, precentral, rostral anterior cingulate, rostral middle frontal and regions of the ventral diencephalon.

Prior brain imaging-based classification models for APTH and PPTH are scarce. We have previously published a classification model based on measures of brain structure and patient clinical features that differentiated PPTH from migraine with a leave-one-out classification accuracy of 78%.^47^ The classification accuracy for differentiating PPTH from HC in this current study was quite high (92%), suggesting that those with PPTH have substantial brain structural changes associated with their underlying brain injuries and persistent post-TBI symptoms. Regions that most contributed to APTH classification included lateral occipital, cuneus, lingual, pericalcarine, superior parietal, precuneus, inferior parietal and cerebellum. Regions contributing to PPTH classification were cerebellum, middle temporal, inferior temporal, inferior parietal, superior parietal, bankssts, precuneus, supramarginal, fusiform white matter, lingual, lateral occipital, postcentral, precentral and posterior cingulate. Several of these regions have been previously demonstrated to differ in structure between those with PTH and HCs including precentral, precuneus, supramarginal, superior and inferior parietal.^48^ Large anterior parietal and temporal opercular regions are reported by Burrowes *et al*.^49^ and Schwedt *et al*.^50^ reported orbitofrontal, supramarginal and superior frontal regions.

This study was not designed to directly compare brain structure between the three different headache groups. However, it is notable that many of the brain regions most contributing to migraine classification are located in the anterior portions of the brain, while many regions most contributing to PTH classification are located more posteriorly. Future studies comparing migraine with PTH should further explore this finding, especially given the multifaceted relationships between the two headache types: (i) PTH often has symptoms that are very similar to migraine; (iii) migraine is a risk factor for developing PTH; and (iii) for those with pre-existing migraine, it can be difficult to differentiate post-injury migraine exacerbation from the development of post-injury PTH.^20,51–53^

The diagnosis of primary headaches and some secondary headaches is currently based on information obtained from a clinical interview. We do not presume that this approach would change in the future, even if highly accurate imaging-based classification models are available. However, classification tasks like those presented in this manuscript are helpful for identifying brain regions that are likely to most contribute to the pathophysiology of migraine and PTH. Furthermore, studies like these set the stage for future investigations that aim to use imaging for differentiating between headache types that have substantial clinical overlap, for identifying new headache subtypes based on imaging data, and perhaps for studies that use imaging data for predicting or tracking patient outcomes.

Limitations of this study include: (i) Smaller headache cohort size than the HC—with larger headache cohort, we expect higher variance of features that would further improve the generalizability of the model. (ii) Although overall our studies included a relatively large number of MRIs, the sample sizes for the validation and independent testing sets were still small. We look forward to testing these classification models further as we collect more brain MRIs from patients who have migraine or PTH and as we collaborate with other research groups collecting similar information. (iii) Patient and HC MRIs were collected from several different scanners using different acquisition parameters. Although this could be seen as a limitation, it can also be interpreted as a strength of our study. It can be argued that the heterogeneity in the data set might reduce the classification accuracy while making the classification results more likely to generalize to new populations of patients. (iv) Some of the brain imaging data included in these studies have been included in prior analyses.^4,5,47,48,50^ Some of the consistency in brain regions that most contributed to classification in this study with findings from our prior studies could be due to the existence of patient overlap between the studies. (v) Although the participants in the IXI data set are considered healthy participants, it is possible that they were not screened for migraine, history of mTBI and PTH. For example, we might assume that about 12% (51 individuals) of the healthy participants had migraine. If some of the participants in this HC data set had migraine or PTH, this would have reduced the accuracy of our classification models; (vi) Older individuals were included in our studies. Although having participants with a broad range of ages could be seen as a strength of the study, it also increases the likelihood of there being undiagnosed comorbidities that could impact brain structure and brain structural changes that occur naturally with age. However, it is unlikely that such situations exerted a major impact on our results since images that contained structural abnormalities that were visible to the human eye were excluded from the analysis and only a small proportion of participants with migraine or PTH were over the age of 60: 4/95 (4.2%) with migraine and 5/97 (5.2%) with PTH. The oldest migraine or PTH participant was 65 years old. (vii) Future studies should investigate DL methodology like that reported herein not only to differentiate those with headache from HCs, but also to help differentiate between headache types, and perhaps for other purposes such as prognosticating patient outcomes. (viii) Information from 14 regions not relevant to our study (including ventricles, vessels, CSF, optic chiasm, choroid plexus and unsegmented white matter) were not included in the analysis, and it is therefore possible that some activation in these regions might have been missed.

## Conclusion

DL approaches are promising for developing migraine and PTH imaging-based classification models. Our studies demonstrated moderate-to-high classification accuracies and identified brain regions that most contributed to classification. Future studies will further validate and fine-tune these models and investigate the possibility of using similar methodologies for building models that differentiate headache types and prognosticate patient outcomes.

## Data Availability

Data from the two studies sponsored by the United States Department of Defense (DOD) (Data sets 3 and 4) and one of the studies sponsored by the National Institutes of Health (NIH) (Data set 5) will be made available through the Federal Interagency Traumatic Brain Injury Research (FITBIR) Informatics System in accordance with the rules and regulations of the DOD and NIH funding contracts. Patient consent for the other NIH-sponsored study (Data set 2) and from the Amgen-sponsored study (Data set 1) did not include a data sharing agreement. The IXI data set (Data set 6) can be obtained from https://brain-development.org/ixi-dataset/.

## Abbreviations

APTH =: acute post-traumatic headache
DL =: deep learning
HC =: healthy control
IXI =: information extraction from images
ML =: machine learning
mTBI =: mild traumatic brain injury
PPTH =: persistent post-traumatic headache
PTH =: post-traumatic headache
